# SIRT1 Expression and Regulation in the Primate Testis

**DOI:** 10.3390/ijms22063207

**Published:** 2021-03-22

**Authors:** Fazal Wahab, Ignacio Rodriguez Polo, Rüdiger Behr

**Affiliations:** 1 Department of Biomedical Sciences, Pak-Austria Fachhochschule: Institute of Applied Sciences and Technology, Mang, Haripur 22620, KP, Pakistan; 2 Platform Degenerative Diseases, German Primate Center, Leibniz Institute for Primate Research, Kellnerweg 4, D-37077 Göttingen, Germany; IRodriguezPolo@dpz.eu

**Keywords:** testis, non-human primate, SIRT1, irisin, marmoset, rhesus

## Abstract

The epigenetic mechanisms controlling germ cell development and differentiation are still not well understood. Sirtuin-1 (SIRT1) is a nicotinamide adenosine dinucleotide (NAD)-dependent histone deacetylase and belongs to the sirtuin family of deacetylases. It catalyzes the removal of acetyl groups from a number of protein substrates. Some studies reported a role of SIRT1 in the central and peripheral regulation of reproduction in various non-primate species. However, testicular SIRT1 expression and its possible role in the testis have not been analyzed in primates. Here, we document expression of SIRT1 in testes of different primates and some non-primate species. SIRT1 is expressed mainly in the cells of seminiferous tubules, particularly in germ cells. The majority of SIRT1-positive germ cells were in the meiotic and postmeiotic phase of differentiation. However, SIRT1 expression was also observed in selected premeiotic germ cells, i.e., spermatogonia. SIRT1 co-localized in spermatogonia with irisin, an endocrine factor specifically expressed in primate spermatogonia. In marmoset testicular explant cultures, *SIRT1* transcript levels are upregulated by the addition of irisin as compared to untreated controls explants. Rhesus macaques are seasonal breeders with high testicular activity in winter and low testicular activity in summer. Of note, *SIRT1* mRNA and SIRT1 protein expression are changed between nonbreeding (low spermatogenesis) and breeding (high spermatogenesis) season. Our data suggest that SIRT1 is a relevant factor for the regulation of spermatogenesis in primates. Further mechanistic studies are required to better understand the role of SIRT1 during spermatogenesis.

## 1. Introduction

Germ cell function is substantially regulated by epigenetic mechanisms including histone modifications [1,2,3]. Histone structure is modified by methylation, phosphorylation, ubiquitination, and acetylation of lysine and serine residues in histone tails of the nucleosome [4]. During histone acetylation, an acetyl group is added to histone tails at lysine residues. Histone acetylation reduces the electrostatic affinity between DNA and histones, resulting in a transcriptionally active euchromatin state of genomic DNA that promotes binding of transcription factors. The reversible actions of histone acetyltransferases (HATs) and histone deacetylases (HDACs) regulate histone acetylation [5,6]. HATs, which comprise the Gcn5/PCAF, p300/CBP, and MYST subfamilies [7], enhance transcription activities in cells by augmenting acetylation of histone lysine residues. In contrast, acetyl groups of histone lysine residues are removed by HDACs, which results in a more compact heterochromatin state limiting binding of transcription factors and hence attenuating transcription [5].

SIRT1, encoded by the *SIRT1* gene, is a HDACs and a member of the sirtuin protein family [8]. SIRT1 causes deacetylation of histones and a number of non-histone proteins. Its activity is dependent on nicotinamide adenosine dinucleotide (NAD) [9]. SIRT1 can deacetylate a large number of different substrates, hence, it plays an important role in a variety of physiological functions, including control of gene expression, metabolism, and aging [8,10,11]. The list of substrates catalyzed by SIRT1 is continuously expanding and includes many different types of transcription factors such as the FoxO family members, the tumor suppressor protein p53, HES1 (hairy and enhancer of split 1), PPARγ (peroxisome proliferator-activated receptor gamma), HEY2 (hairy/enhancer-of-split related with YRPW motif 2), p300, PGC-1α (PPARγ coactivator), CTIP2 (chicken ovalbumin upstream promoter transcription factor (COUPTF)-interacting protein 2), and NF-κB (nuclear factor kappa B) [8,9,10,11,12]. In short, SIRT1 is involved in the regulation of many biological and pathophysiological functions [8,12,13,14,15,16,17,18].

The epigenetic mechanisms affecting germ cell development and differentiation are still not well understood. Recent studies have demonstrated a role of SIRT1 in the central and peripheral regulation of reproduction in various non-primate models [16,17]. It has been documented that complete ablation of *Sirt1* severely compromised reproductive function in both male and female gonads in mice [19]. This defect in reproductive function is caused in part by alterations of the central neuro-endocrine control. Furthermore, specific deletion of *Sirt1* in the male germline also resulted in smaller testes, showing a delay in pre-meiotic germ cell differentiation, reduced number of spermatozoa, higher proportion of spermatozoa with abnormal morphology, and decreased fertility [19]. Nevertheless, testicular SIRT1 expression and its possible role in the testis have not been analyzed in primates. Here, we show SIRT1 expression in testes of different primates and some non-primate species. Moreover, we also analyze the testicular developmental and seasonal changes in expression of SIRT1 in different primate testes. Finally, we tested the effects of exogenous irisin on marmoset testicular tissue in an organotypic culture system [20,21,22].

## 2. Materials and Methods

### 2.1. Study Design

In the present study, we first compared the transcript levels of *SIRT1* in different tissues such as testis, brain (cerebral cortex), heart, skeletal muscle, adipose tissue (collected from abdomen), and liver of adult male monkeys (*n* = 3; common marmoset, *Callithrix jacchus*). SIRT1 expression was then also analyzed by immunohistochemical staining of Bouin-solution-fixed rhesus macaque and common marmoset monkey testicular sections during various developmental (neonatal (1 month), juvenile (5–6 months), and adult (3–5 years)) stages *(n* = 2–5 per stage). Furthermore, testicular SIRT1 expression was determined in additional non-human primate species (e.g., langur (*Trachypithecus selangorensis; n* = 1), baboon (*Papio hamadryas; n* = 3)), and tree shrews (*n* = 3)). The details of testicular samples are provided in Table S1. Moreover, SIRT1 expression was also analyzed in testicular sections of mouse and rat testis at different developmental stages. We also examined the effect of seasonal variations in testicular activity in the adult rhesus monkey [23,24] on testicular *SIRT1* transcript level.

In addition, the effect of irisin (Phoenix pharmaceutical Inc., San Diego, CA, USA) on the *SIRT1* transcript levels in marmoset monkey (*n* = 3) testicular explants was analyzed. For this experiment, the cDNA samples from a previous study were used [20]. These samples were extracted from adult common marmoset testes (*n* = 3) fragments (2 mm diameter, 3 fragments per condition) after incubation with 0.1, 1 and 10 nM irisin concentrations for 6 h in minimal essential medium alpha lacking serum (22561-021, Life Technologies GmbH, Gibco, Darmstadt, Germany) at 35 °C in 5% CO_2_ atmosphere.

### 2.2. Tissue Samples of Nonprimate and Non-Human Primates

Animal care was in accordance with the current regulations as outlined in the animal protection law and reflected in the institutional guidelines. No animal experiments were carried in this study. The testis and other tissues used in the present study were taken from the German Primate Center (DPZ) tissue banks, which include cryo-preserved as well as Bouin-fixed samples. These tissue samples were collected during previously completed studies, which were approved, including ethics, by the competent authorities. 

### 2.3. Immunohistochemistry on Sectioned Tissues

For fixation, tissues were put in Bouin’s solution overnight. Subsequently, these tissues were kept for two days in 70% ethanol before embedding in paraffin. For immunohistochemistry, 5 µm sections, which were cut from embedded tissues, were placed on slides. These tissue sections were deparaffinized in xylene and rehydrated by passing through an ethanol gradient series and finally put in water. For retrieval of the antigen, these tissue sections were heated in citrate buffer (10 mM) for 10 min in a microwave oven. After cooling down and washing in wash buffer, the sections were put in a peroxidase blocking reagent for half hour at ambient temperature (DakoCytomation Carpinteria, CA, USA). Subsequently, the tissue sections were incubated overnight with SIRT1 (HPA006295, Sigma Aldrich) and irisin (H-067-17, Phoenix Pharmaceuticals, CA, USA) primary antibodies at 4 °C. After washing, the tissue sections were incubated at room temperature for 30 min first with secondary antibody and subsequently with horse-radish peroxidase (HRP)-conjugated streptavidin (DakoCytomation Universal kit). After proper washing in wash buffer, the testicular sections were incubated for 5 to 15 min with 3,3′-diaminobenzidine, the chromogenic HRP substrate. The counterstaining of tissue sections was done with Mayer’s hematoxylin. A Zeiss Axioskop microscope was used to take images. The double staining of SIRT1 and irisin was carried out with the Envision double stain system (DakoCytomation, Cat #K5361). 

### 2.4. Extraction and Quantification of RNA 

The NucleoSpin RNA-plus kit (Macherey-Nagel GmbH, Germany) was utilized for total RNA extraction from the tissue samples as per the manufacturer’s instructions. Genomic DNA contamination was removed by the DNA-free™ kit (AM1906, Ambion, Life Technologies, Waltham, MA, USA). 

After measuring RNA concentrations via a Nanodrop spectrophotometer (ThermoScientific, Wilmington, DE, USA), 1 µg of total RNA was converted to cDNA using the Omniscript RT kit 200 (Qiagen) along with negative controls (-RT). The synthesized cDNA was diluted to a 15 ng/μL concentration and frozen at −20 °C until quantification. The real-time qPCR analysis was carried out using a StepOnePlus qPCR System (Applied Biosystems, Thermo Fisher Scientific, Waltham, MA, USA). The details of the qPCR reaction were published previously [20]. Briefly, each qPCR amplification reaction was done in triplicate in the StepOnePlus System (Applied Biosystems, Carlsbad, CA, USA) with the SybrGreen MasterMix. The sequences of the primers used in qPCR are provided in Table S2. The primers were selected by their melting curve and efficiency. The 2^−ΔΔCT^ method was used for the calculation of the relative mRNA levels.

### 2.5. Statistics

GraphPad Prism statistical software was used to perform all statistical analyses. The Student’s *t*-test and ANOVA with the post hoc Tukey test were used to compare two and multiple groups, respectively. All data is shown as means ± SEM. The statistical significant was set at *p* value of <0.05.

## 3. Results

### 3.1. Tissue Distribution of SIRT1 Transcripts 

**Figure 1:**
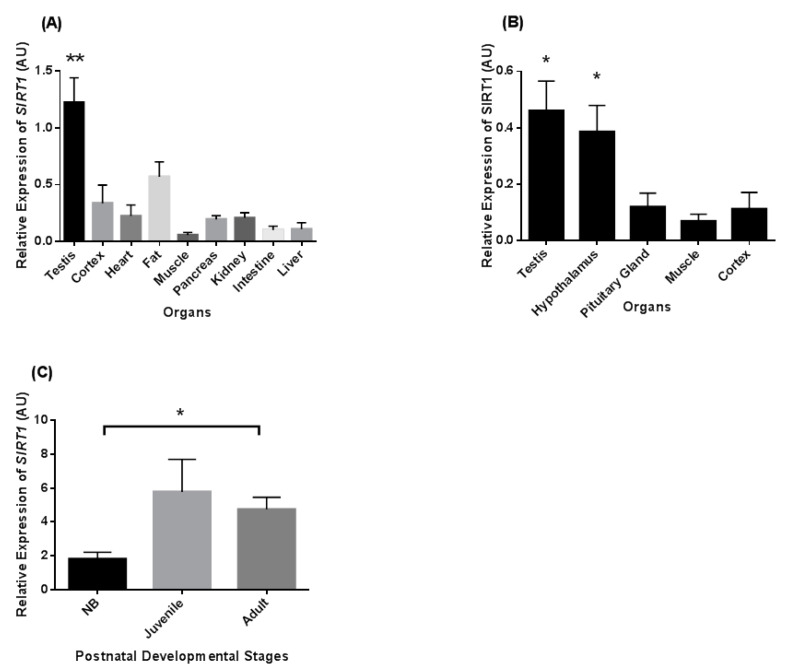
Sirtuin-1 (*SIRT1*) relative transcript levels in different tissues and during postnatal (neonatal (up to 1 month), juvenile (5–6 months), and adult (3–5 years)) stages of the common marmoset monkey. (**A**) The transcript levels of *SIRT1* is highest in testis as compared to other tissues. (**B**) *SIRT1* mRNA was highest in testis and hypothalamus as compared to pituitary gland, muscle, and cortex tissues. (**C**) The expression level of *SIRT1* mRNA was significantly higher in adult as compared to the neonatal stage. * *p* < 0.05, ** *p* < 0.01 significant increase.

We compared abundances of *SIRT1* mRNA in the brain, adipose tissue, heart, kidney, pancreas, skeletal muscle, intestine, liver, and testis of common marmoset monkeys (*n* = 3 adult males). Testicular *SIRT1* levels were highest in comparison to the other tissues (Figure 1A). *SIRT1* mRNA levels were in the medium range in fat, cortex, pancreas, and kidney as compared to the testis and other organ levels. Comparison of *SIRT1* mRNA expression in various components of the reproductive axis showed that its transcript levels were high in both testis and hypothalamus as compared to pituitary gland and control tissues such as muscle and cortex (Figure 1B).

Relative levels of *SIRT1* mRNA expression during three postnatal developmental stages of marmoset testes are shown in Figure 1C. The relative *SIRT1* mRNA levels (*p* < 0.05) increased from the neonatal to the adult stage. The *SIRT1* mRNA levels were also higher in the juvenile stage as compared to newborn (NB), but no statistical significance was noted.

### 3.2. SIRT1 Expression in the Non-Human Primates Testes

**Figure 2:**
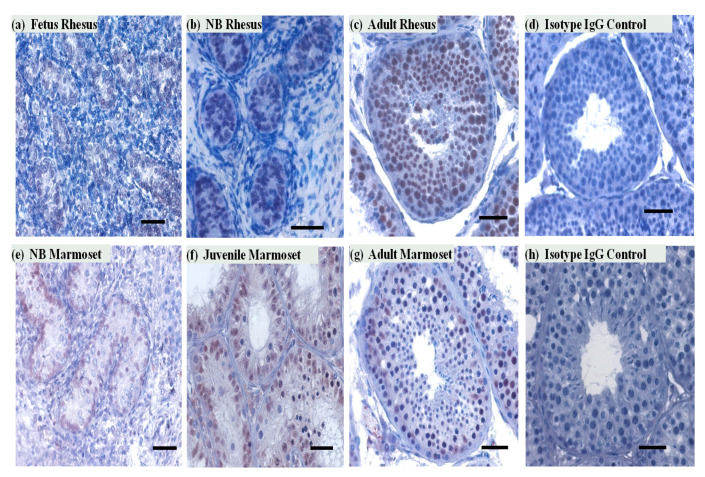
SIRT1 expression in rhesus and marmoset monkey testes during different developmental stages. (**a**,**b**) Very few SIRT1-positive cells were observed in fetal and NB (1 month) rhesus monkey testes in the seminiferous tubules. (**c**,**d**) In the adult rhesus testis, all seminiferous tubule cross sections contained many SIRT1 positive germ cells. No staining was noted in control Immunoglobulin (IgG) antibody incubated testicular cross section of rhesus monkeys. (**e**,**f**) In NB (5 days) and juvenile (5–6 months) marmoset testes, SIRT1 positive cells were mainly observed in the tubular compartment. No staining was observed with control antibody (isotypic IgGs). (**g**,**h**) In the adult common marmoset testis SIRT1 was detected in germ and Sertoli cells. Scale bars, 50 μm.

To determine the cell-type-specific expression of SIRT1 protein in the testis, we carried out immunohistochemical staining of testicular sections of NHP. In the marmoset monkey testis, SIRT1 expression was observed in both Sertoli and germ cells. In the adult rhesus testis, immunoreactivity of SIRT1 was mainly noted in germ cells. Most of the meiotic and postmeiotic germ cells were SIRT1 positive. SIRT1 expression was also observed on some selected pre-meiotic germ cells (Figure 2). SIRT1 signals in seminiferous tubules were observed in all developmental stages in both rhesus and marmoset monkeys testes sections.

**Figure 3:**
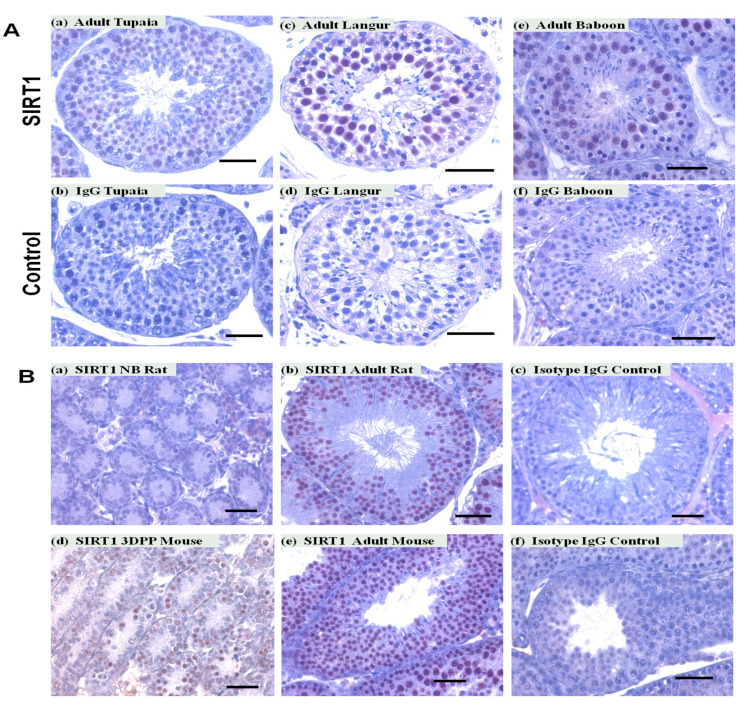
SIRT1 expression in testes of different primates and non-primates species. SIRT1 expression was observed in cells of the tubular compartment of tupaia (**A**) a, langur (**A**) c, baboon (**A**) e, rat (**B**) a,b, and mouse (**B**) d,e testes. No staining was noted in IgG controls. Scale bars, 50 μm.

Additionally, SIRT1-positive germ cells were also observed in adult testes of the langur (*Semnopithecus johnii),* baboon (*Papio hamadryas*), and Tupaia (Figure 3A(a–f)). In addition to primates, we have also noted SIRT1-positive germ cells in rodents (mouse and rat) (Figure 3B(a–f)).

### 3.3. SIRT1 mRNA and Protein Expression in Rhesus Monkey Testis during Various Seasons

**Figure 4:**
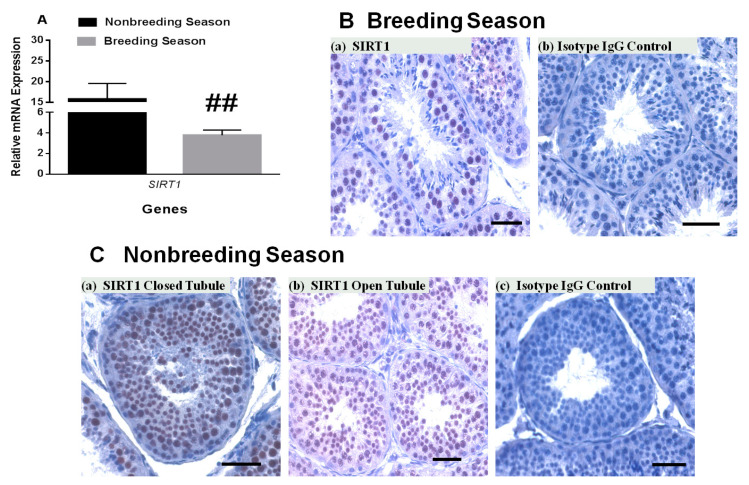
Comparison of *SIRT1* / SIRT1 expression in breeding and nonbreeding seasons. (**A**) *SIRT1* mRNA expression was significantly decreased during the breeding season as compared to nonbreeding season in testes of the adult rhesus monkeys testes (*n* = 3–5 per season). (**B**,**C**) The number of SIRT1-positive germ cells was low in the breeding season (**B**) a,b as compared to the nonbreeding season (**C**) a–c. Scale bar, 50 μm.

It is well-established that seminiferous tubules of the rhesus monkey show changes in response to seasonal variations [23,24]. During the breeding season, most of the seminiferous tubules have elongated spermatids and are open. In contrast, during the nonbreeding season, many of the seminiferous tubules do not show full spermatogenesis with several tubules without a clear lumen. The relative *SIRT1* transcript level was decreased to ~30% in rhesus monkey testes during the breeding season (January) compared to the nonbreeding season (May–July, Figure 4A). SIRT1-positive germ cells were present in both closed and open seminiferous tubules in the nonbreeding season as well as breeding season. Mature spermatids lack SIRT1.

### 3.4. Exogenous Irisin Effect on SIRT1 Transcript Levels in Testis 

**Figure 5:**
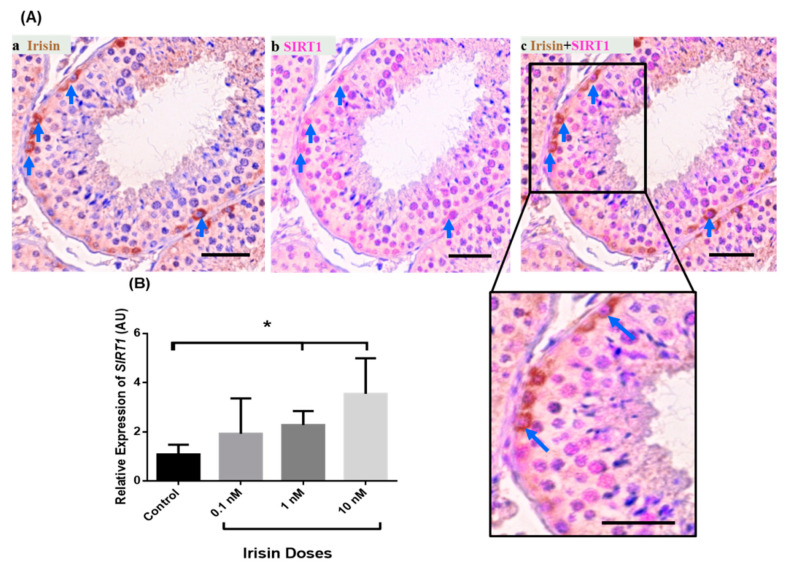
Double staining of SIRT1 and irisin on marmoset testis sections. (**A**) Irisin-positive spermatogonia also showed SIRT1 expression. (**B**) The effect of exogenous irisin on testicular *SIRT1* transcript abundance in organotypic testis cultures. One and 10 nM irisin application to cultured marmoset testicular explants significantly increased the transcript levels of *SIRT1* as compared to the control (PBS treated testicular explants). * *p <* 0.05 significant increase.

Irisin is an endocrine- and metabolic-state-related factor and is present in the primate testis in undifferentiated spermatogonia [20]. SIRT1 co-staining with irisin demonstrated that some irisin positive cells are also SIRT1 positive (Figure 5A, Supplementary Figure S1). To check whether irisin exerts an effect on *SIRT1* expression in testis of the marmoset monkey, we carried out an organotypic culture of testis fragments [22] and determined the relative *SIRT1* transcript abundance in testis fragment cultures after incubation with different concentrations of irisin (0.1, 1, and 10 nM/mL). Irisin exerts a dose-dependent effect on *SIRT1* mRNA levels. There was only a slight increase in response to the 0.1 nM/mL dose of irisin. In contrast, there was a significant augmentation of *SIRT1* expression in response to 1 and 10 nM irisin compared to the untreated control (Figure 5B).

## 4. Discussion

Epigenetic factors play key roles in basically all biological processes in the mammalian body, including testicular devolvement and function [1,2,3]. However, epigenetic mechanisms affecting germ cell development and differentiation are still not very well understood. Recent studies have demonstrated a role of SIRT1 deacetylase in central and peripheral regulation of reproduction in various non-primate models [16,17]. Nevertheless, there is a scarcity of data on testicular SIRT1 expression and its potential role in primate reproductive function. In the present study, we demonstrate expression of SIRT1 in the testes of different primate species. SIRT1 is expressed mainly in the cells of seminiferous tubules, particularly in germ cells. The majority of SIRT1-positive germ cells were in the meiotic and postmeiotic phase of differentiation. However, SIRT1 expression was also observed in selected premeiotic germ cells, i.e., spermatogonia. SIRT1 was co-localized in some spermatogonia with irisin, an endocrine factor also expressed in primate spermatogonia [20]. Importantly, *SIRT1* transcript levels were significantly increased in marmoset testicular explant cultures in response to addition of irisin as compared to untreated controls explants. Rhesus macaques are seasonal breeders with high testicular activity in winter and low testicular activity in summer [23,24]. Interestingly, both *SIRT1* mRNA and SIRT1 protein levels are altered between nonbreeding (low spermatogenesis) and breeding (high spermatogenesis) seasons. Our data suggest that SIRT1 might be an important factor for the regulation of spermatogenesis in the primate testes. However, mechanistic studies are needed to decipher the role of SIRT1 in the primate testis.

Interestingly, in the marmoset testis immunohistochemical SIRT1 signals were also encountered in the nuclei of Sertoli cells. Sertoli cell staining was seen throughout the cycle of the seminiferous tubule. In the testes of all other species examined, no SIRT1 signals were seen in Sertoli cells. Possible explanations for this finding may include alternative splicing in Sertoli cells resulting in differential detectability of the protein or repurposing of promoters resulting in novel gene expression patterns in different cells types in different species. Currently, ongoing single nuclei transcript sequencing of testes of different primate and rodent species will provide additional information on differential *SIRT1* gene expression in the male mammalian gonad.

We noticed high SIRT1 immunoreactivity in the nucleus of meiotic and postmeiotic germ cells. However, there were some SIRT1-positive premeiotic germ cells (spermatogonia) as well. Acetylation and deacetylation activities play a key role in proper execution of meiosis [5,7]. Strong expression of SIRT1 in meiotic cells suggests an important role during meiosis. Indeed, SIRT1 ablation, in the whole body or specifically in germ cells in mice, leads to a complete loss of quantitatively and qualitatively normal spermatogenesis [16,19]. These mice exhibit a greatly reduced spermatogenesis or even a Sertoli cell only syndrome [19]. This finding, however, suggests that SIRT1 already has an important function in premeiotic germ cell stages. Future studies on the role of SIRT1 are important to unveil its potential roles during the different phases of spermatogenesis. 

Irisin is an endocrine- and metabolic-state-dependent factor and is also expressed in the testis [20]. Irisin application to an adult marmoset monkey testicular fragment culture causes an increase in the expression of *SIRT1* transcript levels. Previously, some studies documented a role for SIRT1 in testicular physiology [16,17]. However, the interplay between irisin and SIRT1 in the testis needs scholarly investigation. Nevertheless, our results show that irisin is possibly involved in SIRT1 synthesis. 

Previous studies documented seasonal variations in the seminiferous tubules of rhesus monkeys [23,24]. These changes in testicular physiology provide an interesting model system for investigations concerning initiation of spermatogenesis and particularly proliferation of spermatogonia [23,24]. Many of the seminiferous tubules are closed during the nonbreeding season, and spermatogenesis occurs only at a reduced level. During the breeding season, the vast majority of the seminiferous tubules are open and spermatogenic output is high. Both *SIRT1* and SIRT1 expression is changed during the nonbreeding and breeding seasons in testes of the adult rhesus monkey. In the nonbreeding season with low spermatogenic activity, the expression of *SIRT1* was high. However, when spermatogenesis is active during the breeding season, expression of *SIRT1* is low and numbers of SIRT1-positive cells are low (Supplementary Figure S2). These findings show a negative correlation of spermatogenesis and SIRT1 expression. Our findings suggest that increased deacetylation activities of SIRT1 during the nonbreeding season might be involved in down-regulation of spermatogenesis. In contrast, low SIRT1 activity during the breeding season might lead to increased spermatogenesis. In short, SIRT1-mediated deacetylation-regulated signaling might contribute to the molecular cascades that change the spermatogenic state in primates and possibly other mammalian species. 

In summary, SIRT1 is expressed in primate testes of different non-human primate species as well as in the tree shrew. Relative *SIRT1* expression levels are altered during development and seasonal variations. The role of SIRT1-induced deacetylation in the regulation of spermatogenesis and the in vivo relevance of the effect of irisin on *SIRT1* expression in the testis need to be investigated in future studies.

## Data Availability

Not applicable.

## Supplementary Materials

The following are available online at https://www.mdpi.com/1422-0067/22/6/3207/s1, Figure S1: Comparison of Irisin-, SIRT1-, and Irisin+SIRT1 Germ Cells in 15 seminiferous tubules of common marmoset testes, Figure S2: Comparison of SIRT1 positive Germ Cells in breeding (*n* = 2) and nonbreeding (*n* = 3) season in open (A) and closed (B) seminiferous tubules in rhesus monkey testes. The number of SIRT1 positive Germ Cells significantly decreases in breeding season as compared to nonbreeding season, Table S1: Details of the species and number of animals used for analysis, Table S2: List of primers used for real-time qPCR with Ensemble transcript ID/ accession number.

## References

[1] Song N., Endo D., Koji T. (2014). Roles of epigenome in mammalian spermatogenesis. Reprod. Med. Biol..

[2] Eun S.H., Gan Q., Chen X. (2010). Epigenetic regulation of germ cell differentiation. Curr. Opin. Cell Biol..

[3] Meikar O., Da Ros M., Kotaja N. (2013). Epigenetic regulation of male germ cell differentiation. Subcell Biochem..

[4] Bird A. (2007). Perceptions of epigenetics. Nature.

[5] Shahbazian M.D., Grunstein M. (2007). Functions of site-specific histone acetylation and deacetylation. Annu. Rev. Biochem..

[6] Ma P., Pan H., Montgomery R.L., Olson E.N., Schultz R.M. (2012). Compensatory functions of histone deacetylase 1 (HDAC1) and HDAC2 regulate transcription and apoptosis during mouse oocyte development. Proc. Natl. Acad. Sci. USA.

[7] Gu L., Wang Q., Sun Q.Y. (2010). Histone modifications during mammalian oocyte maturation: Dynamics, regulation and functions. Cell Cycle.

[8] Michan S., Sinclair D. (2007). Sirtuins in mammals: Insights into their biological function. Biochem. J..

[9] Pillarisetti S. (2008). A review of Sirt1 and Sirt1 modulators in cardiovascular and metabolic diseases. Recent Pat. Cardiovasc. Drug Discov..

[10] Yamamoto H., Schoonjans K., Auwerx J. (2007). Sirtuin functions in health and disease. Mol. Endocrinol..

[11] Haigis M.C., Guarente L.P. (2006). Mammalian sirtuins—Emerging roles in physiology, aging, and calorie restriction. Genes Dev..

[12] Kosciuk T., Wang M., Hong J.Y., Lin H. (2019). Updates on the epigenetic roles of sirtuins. Curr. Opin. Chem. Biol..

[13] Chianese R., Viggiano A., Urbanek K., Cappetta D., Troisi J., Scafuro M., Guida M., Esposito G., Ciuffreda L.P., Rossi F. (2018). Chronic exposure to low dose of bisphenol A impacts on the first round of spermatogenesis via SIRT1 modulation. Sci. Rep..

[14] D’Angelo S., Mele E., Di Filippo F., Viggiano A., Meccariello R. (2021). Sirt1 Activity in the Brain: Simultaneous Effects on Energy Homeostasis and Reproduction. Int. J. Environ. Res. Public Health.

[15] Liu C., Song Z., Wang L., Yu H., Liu W., Shang Y., Xu Z., Zhao H., Gao F., Wen J. (2017). Sirt1 regulates acrosome biogenesis by modulating autophagic flux during spermiogenesis in mice. Development.

[16] Tatone C., Di Emidio G., Barbonetti A., Carta G., Luciano A.M., Falone S., Amicarelli F. (2018). Sirtuins in gamete biology and reproductive physiology: Emerging roles and therapeutic potential in female and male infertility. Hum. Reprod. Update.

[17] Vazquez M.J., Toro C.A., Castellano J.M., Ruiz-Pino F., Roa J., Beiroa D., Heras V., Velasco I., Dieguez C., Pinilla L. (2018). SIRT1 mediates obesity- and nutrient-dependent perturbation of pubertal timing by epigenetically controlling Kiss1 expression. Na.t Commun..

[18] Wu L., Zhang A., Sun Y., Zhu X., Fan W., Lu X., Yang Q., Feng Y. (2012). Sirt1 exerts anti-inflammatory effects and promotes steroidogenesis in Leydig cells. Fertil. Steril..

[19] Bell E.L., Nagamori I., Williams E.O., Del Rosario A.M., Bryson B.D., Watson N., White F.M., Sassone-Corsi P., Guarente L. (2014). SirT1 is required in the male germ cell for differentiation and fecundity in mice. Development.

[20] Wahab F., Drummer C., Matz-Rensing K., Fuchs E., Behr R. (2020). Irisin is expressed by undifferentiated spermatogonia and modulates gene expression in organotypic primate testis cultures. Mol. Cell. Endocrinol..

[21] Medrano J.V., Vilanova-Perez T., Fornes-Ferrer V., Navarro-Gomezlechon A., Martinez-Triguero M.L., Garcia S., Gomez-Chacon J., Povo I., Pellicer A., Andres M.M. (2018). Influence of temperature, serum, and gonadotropin supplementation in short- and long-term organotypic culture of human immature testicular tissue. Fertil. Steril..

[22] Kristensen D.M., Desdoits-Lethimonier C., Mackey A.L., Dalgaard M.D., De Masi F., Munkbol C.H., Styrishave B., Antignac J.P., Le Bizec B., Platel C. (2018). Ibuprofen alters human testicular physiology to produce a state of compensated hypogonadism. Proc. Natl. Acad. Sci. USA.

[23] Bansode F.W., Chowdhury S.R., Dhar J.D. (2003). Seasonal changes in the seminiferous epithelium of rhesus and bonnet monkeys. J. Med. Primatol..

[24] Higashi Y., Takahashi J., Yoshida K., Winters S.J., Oshima H., Troen P. (1984). Seasonal changes in steroidogenesis in the testis of the rhesus monkey (*Macaca mulatta*). J. Androl..

