# Supplementary material for: SIRT1 Expression and Regulation in the Primate Testis
Source: Int J Mol Sci. 2021 Mar 22;22(6):3207. doi: 10.3390/ijms22063207 (PMC8004242; doi:10.3390/ijms22063207)
Supplement: Supplementary file 1 [file ijms-22-03207-s001.pdf]

SIRT1 Expression and Regulation in the Primate Testis

Fazal Wahab, Ignacio Rodriguez Polo and Rüdiger Behr

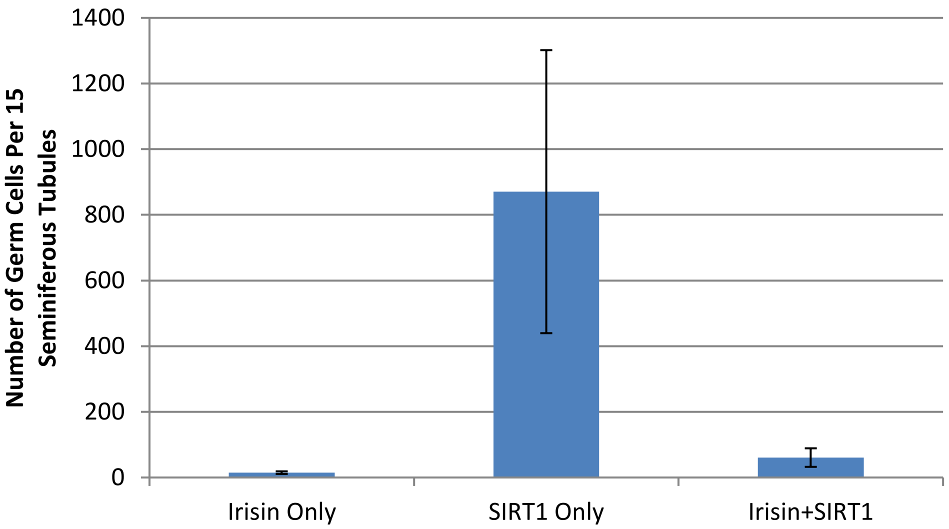

Figure S1. Comparison of Irisin-, SIRT1-, and Irisin+SIRT1 Germ Cells in 15 seminiferous tubules of common marmoset testes.

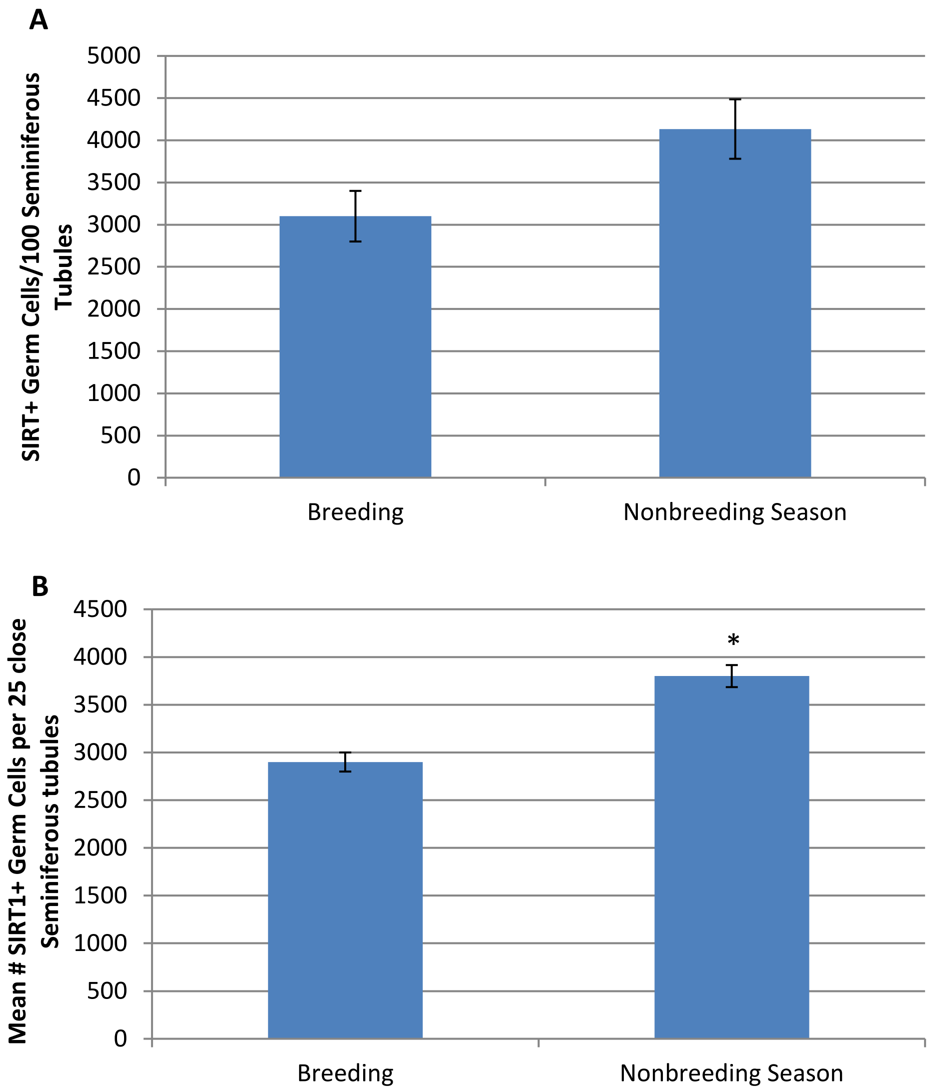

Figure S2. Comparison of SIRT1 positive Germ Cells in breeding (n = 2) and nonbreeding (n = 3) season in open (**A**) and closed (**B**) seminiferous tubules in rhesus monkey testes. The number of SIRT1 positive Germ Cells significantly decreases in breeding season as compared to nonbreeding season.

Supplementary Table S1. Details of the species and number of animals used for analysis.

Species	Stage	Number of Animals
Marmoset	New born, juvenile, adult	3-5 per stage
Rhesus monkey	Fetal, NB, adult	2-6 per stage
Baboon	Adult	3
Langur	Adult	1
Tree Shrew	Adult	3

Supplementary Table S2. List of primers used for real time qPCR with Ensemble transcript ID/ accession number.

**Gene**	**Transcript ID/Accession#**	**Primer**	**Sequences (5'-3')**
SIRT1-Marmoset	XM_002756312.4	Forward<br>Reverse	CACTAATTCCAAGTTCTATACC<br>TATTCACCACCTAACCTATG
GAPDH-Marmoset	DD279474	Forward<br>Reverse	TCGGAGTCAACGGATTTGGTC<br>TTCCCGTTCTCAGCCTTGAC
18S rRNA-Marmoset	AB571241	Forward<br>Reverse	CGCGGTTCTATTTTGTTGGT<br>AGTCGGGCATCGTTTATGGTC
GAPDH-Rhesus	XM_001105471.1	Forward<br>Reverse	GAAATCCCATCACCATCTTCCAGG<br>GAGCCCCAGCCTTCTCCATG
ACTB-Rhesus	NM_001033084.1	Forward<br>Reverse	CCTGGCACCCAGCACAAT<br>GGGCCGGACTCGTCATAC
SIRT1-Rhesus	XM_015147517.2	Forward<br>Reverse	TATGCTCGCCTTGCTGTAG<br>AACTTGAAGAATGGTCTTGGAT
